# TableBorderNet: A Table Border Extraction Network Considering Topological Regularity

**DOI:** 10.3390/s25133899

**Published:** 2025-06-23

**Authors:** Jing Yang, Shengqiang Zhou, Xialing Li, Yuchun Huang, Honglin Jiang

**Affiliations:** 1 School of Remote Sensing and Information Engineering, Wuhan University, Wuhan 430079, China; jingyjq@foxmail.com (J.Y.); 2023202130071@whu.edu.cn (X.L.); 2 China Railway Changjiang Transport Design Group Co., Ltd., Chongqing 401121, China; zhousq616@163.com (S.Z.); 117782315773@163.com (H.J.)

**Keywords:** table border extraction, deep learning, semantic segmentation, topology-aware learning

## Abstract

Accurate extraction of table borders in scanned road engineering drawings is crucial for the digital transformation of engineering archives, which is an essential step in the development of intelligent infrastructure systems. However, challenges such as degraded borders, image blur, and character adjoining often hinder the precise delineation of table structures, making automated parsing difficult. Existing solutions, including traditional OCR tools and deep learning methods, struggle to consistently delineate table borders in the presence of these visual distortions and fail to perform well without extensive annotated datasets, which limits their effectiveness in real-world applications. We propose TableBorderNet, a semantic segmentation framework designed for precise border extraction under complex visual conditions. The framework captures structural context by guiding convolutional feature extraction along explicit row and column directions, enabling more accurate delineation of table borders. To ensure topological consistency in complex or degraded inputs, a topology-aware loss function is introduced, which explicitly penalizes structural discontinuities during training. Additionally, a generative self-supervised strategy simulates common degradation patterns, allowing the model to achieve strong performance with minimal reliance on manually annotated data. Experiments demonstrate that the method achieves an Intersection-over-Union of 94.2% and a topological error of 1.07%, outperforming existing approaches. These results underscore its practicality and scalability for accelerating the digitization of engineering drawings in support of data-driven road asset management.

## 1. Introduction

Tables serve as essential carriers of structured data across various industries including finance, healthcare, and civil engineering. In road engineering, tables play a critical role in storing and conveying key information within design drawings, project budgets, and construction schedules. These tables typically contain data such as material inventories, construction procedures, and timelines, all of which directly influence project planning and implementation. Despite their significance, the processing of engineering tables remains a largely manual process involving scanning the tables into electronic formats followed by labor-intensive and error-prone manual transcription.

Tables in road engineering drawings exhibit domain-specific characteristics that distinguish them from those in general document contexts. While they typically follow standardized design conventions with regular row-column structures and explicit gridlines, several challenges complicate their automated extraction. First, degraded borders—caused by environmental wear, aging, or scanning imperfections—often result in broken or blurred gridlines that hinder structural recognition. Second, character adjoining and background interference frequently occur when text elements merge with borders or the background during digitization, disrupting the visual distinction between content and structure. Third, the scale of infrastructure projects demands high-throughput processing, rendering manual digitization impractical and emphasizing the necessity for robust and automated border extraction solutions.

**Figure 1:**
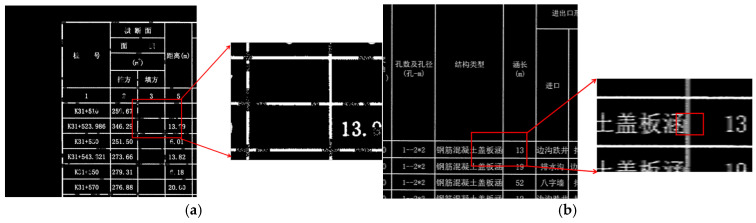
Tables With Structural Anomalies. The Chinese characters in the table are all professional terms related to road engineering. (**a**) Discontinuous Borders. (**b**) Borders Joined with Characters.

**Figure 2:**
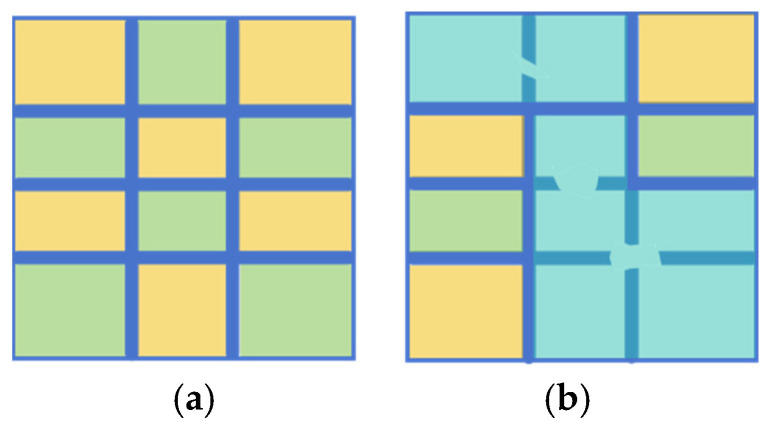
Connected Component Analysis of Table Borders. Different colors of adjacent blocks denote the absence of connectivity between regions, while the same color denotes the presence of connectivity. (**a**) Complete borders. (**b**) Borders with topological defects.

As illustrated in Figure 1, degraded road engineering tables present various structural anomalies. Figure 1a displays table borders with evident discontinuities, while lines remain visible in some regions, they are interrupted or blurred in others. These discontinuous lines are vulnerable to image noise and may impair the differentiation of adjacent table cells, resulting in region localization errors. To further illustrate the impact of border discontinuities on table structure, we conducted a connected component analysis of the detected gridlines. In an ideal scenario, as depicted in Figure 2a, each complete table border is represented by a single, fully connected component, allowing for accurate cell-region localization. However, when faced with discontinuities, as shown in Figure 2b, segments of the border can merge into fewer, larger components, leading to topological defects. This merging complicates the distinction between adjacent cells and hinders effective extraction of tabular data. Figure 1b depicts borders with unclear edges caused by low local contrast between the borders and background, as well as by character-gridline overlap, where text partially adjoins with the borders. This blending typically necessitates additional preprocessing, which increases computational complexity and may introduce further errors.

However, most existing studies on table recognition primarily focus on well-structured and visually clear table images, which present relatively low levels of difficulty and fail to account for the aforementioned irregularities commonly found in road engineering tables [1,2]. Some traditional methods do incorporate topological structure information of tables, but their hand-crafted algorithms often lack generalizability [3,4]. On the other hand, most deep learning-based approaches rely on conventional semantic segmentation architectures, without explicitly modeling the unique structural characteristics of tables [5,6]. To address these limitations, this study proposes TableBorderNet, a neural network designed for complete extraction of table topological structures from scanned road engineering drawings. The design of TableBorderNet incorporates three core components: SelfSynth Training, SliceConv Enhancement and TopoLoss. Together, these components leverage the topological regularity of table structures in scanned drawings to enhance the completeness and robustness of structural recognition. The key innovations of this work are as follows: (1) SelfSynth Training: A generative self-supervised training strategy that synthesizes defective table samples from complete ones, thereby reducing the cost of manual annotation and enabling topological extraction without labeled data. This strategy significantly lowers the sample dependency of the extraction network and enhances adaptability to diverse table defect scenarios without necessitating changes to the network structure. (2) SliceConv Enhancement: A network architecture for table structure extraction that includes a topology feature enhancement module using row-column slice-by-slice convolution and feature aggregation to expand contextual awareness for accurate border extraction. (3) TopoLoss: A topology-aware loss function and corresponding evaluation metrics that assess and guide correction of topological deficiencies in the predicted borders, thereby improving extraction completeness.

The rest of this paper is organized as follows: Section 2 reviews related work in table border extraction and document analysis. Section 3 details the TableBorderNet methodology and architectural components. Section 4 presents extensive experimental results across various degradation scenarios. Section 5 concludes with insights and future research directions.

## 2. Related Work

Traditional approaches to table structure recognition primarily rely on image processing and pattern recognition techniques that exploit geometric features—such as lines, gaps, and edges—to identify structural components of tables. Among these, methods based on line feature detection, such as the Hough transform [7], constitute a classic framework. The Hough transform is used to detect straight lines in an image, facilitating the identification of rows and columns by analyzing the presence and orientation of horizontal and vertical lines. However, this approach is highly contingent on binary segmentation quality and sensitive to edge blurring or noise, often leading to inaccurate line extraction—particularly in positioning—which undermines subsequent semantic information mapping based on table structures. Although conventional projection or Hough-based statistical methods effectively leverage macroscopic border features, their performance degrades with increasing table size: larger table areas amplify fluctuations in single-pixel line extraction, weakening interference resistance and limiting applicability to the diverse table sizes and styles present in road engineering drawings. The reliance on heuristic rules tailored to specific data characteristics further highlights the limitations of traditional methods, which are typically effective only on well-structured, high-quality data and struggle to handle complex scenarios such as incomplete or ambiguous table boundaries [8].

With the rapid advancement of deep learning, researchers have increasingly applied these techniques to table structure extraction. Deep learning offers the advantage of automatic feature learning, demonstrating superior adaptability compared to traditional methods—especially in scenarios involving blurred or disturbed table borders. Current deep learning-based approaches can be broadly categorized into object detection [9,10,11,12,13,14] and semantic segmentation [15,16,17,18] methodologies. Object detection methods adopt a bottom-up strategy, reconstructing table structures starting from individual cells. For example, Qiao L et al. [19] framed table structure detection as an object detection task, employing Mask R-CNN [20] to detect cells and text regions, derive local and global boundaries, and restore structures through post-processing rules like cell matching and merging. However, this reliance on accurate text block or cell detection renders it ill-suited for complex tables with composite cells formed by varied merging strategies. Conversely, Paliwal et al. [21] proposed TableNet, a top-down approach that first localizes the entire table, then partitions cells to extract structure. By integrating the semantic segmentation encoder model of Long et al. [22] to generate masks for table and column regions, and applying predefined row segmentation rules, TableNet reconstructs global structures. However, these methods are predominantly designed for standard table data and fail to account for the complexities encountered in more challenging scenarios. Wang et al. [6] addressed a variety of table types in their study, including paper documents, advertisements, street announcements, and merchandise packaging, and considered potential issues such as distorted table lines. Nonetheless, their approach did not address the problem of indistinct or missing boundaries, limiting its applicability to well-defined tables. Moreover, most of the aforementioned methods are built upon general-purpose deep learning architectures commonly used in computer vision, without incorporating the unique structural properties of tables. As a result, the segmentation outputs often fall short in supporting downstream tasks. For example, TableSegNet [23] demonstrates that standard semantic segmentation networks may fail to distinguish the boundaries between adjacent tables, leading to merged table regions and incorrect cell extraction. To achieve more complete table extraction and facilitate subsequent sub-cell segmentation, Pang et al. [24] proposed TableRocket, which emphasizes the integrity of segmentation after identifying cells. However, their strategy remains effective only for simple and clearly structured tables.

In contrast to existing approaches, our TableBorderNet explicitly targets the topological integrity of table borders, a critical factor for accurate cell delineation and subsequent information extraction. By combining topology feature enhancement, topology-aware loss functions, and self-supervised learning, we address the fundamental limitations of current methods when applied to degraded engineering drawings.

## 3. Methodology

**Figure 3:**
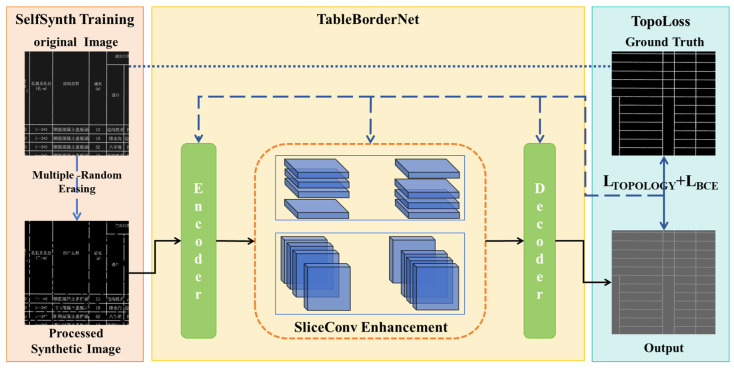
Structure of TableBorderNet. The Chinese characters in the table are all professional terms related to road engineering.

Utilizing the strong structural priors present in bordered tables from road engineering drawings, this study reformulates the table structure extraction task as a topological semantic segmentation problem, focusing on robust border delineation under challenging conditions. The overall framework, illustrated in Figure 3, is composed of three key components: SelfSynth Training, SliceConv Enhancement, and TopoLoss, which together address the typical degradation patterns and structural requirements of engineering tables. As shown in the left part of Figure 3, the SelfSynth Training module applies a Multiple-Random-Erasing strategy to the original table images, simulating common table border defects such as partial erasure, blur, and discontinuities, thereby enhancing the model’s robustness to real-world degradations and enabling it to better handle imperfect inputs. The middle section illustrates SliceConv, a structure-guided module that extracts row- and column-wise contextual feature aggregation to capture explicit structural patterns of bordered tables, thereby enabling more accurate and complete border segmentation guided by the structural prior of bordered tables. Finally, the right part incorporates TopoLoss, a topology-aware loss function that penalizes structural discontinuities during training, encouraging the model to preserve the global table layout even in degraded inputs and ensuring topological consistency.

### 3.1. SelfSynth Training

**Figure 4:**
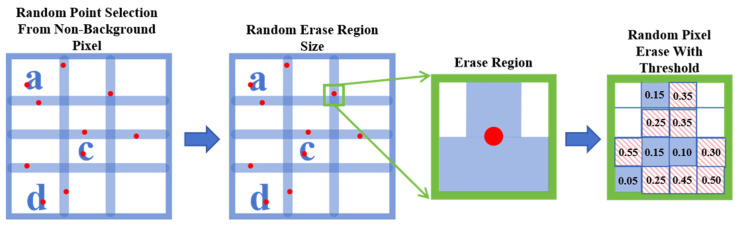
Illustration of SelfSynth Multiple-Random Erasing for Self-supervised Dataset Generation: In the first panel, red dots represent randomly selected points, and the letters a, c, d refer to the text within the table; in the last panel, the blue background designates the regions to be preserved, and the red diagonal lines on a white background indicate the regions to be erased.

The automatic extraction of table borders from scanned engineering drawings is often challenged by the presence of various degradation artifacts inherent to the digitization process. These imperfections tend to obscure structural information and introduce inconsistencies in pixel-level annotations, complicating both the annotation process and the training of reliable models. Moreover, manually labeled datasets rarely reflect the full range of real-world variations, limiting the model’s generalization capacity. To enhance robustness under such conditions, a self-supervised synthesis strategy SelfSynth is introduced to simulate representative forms of degradation, allowing the network to learn topological features of degradations with no need for laborious annotation. As shown in Figure 4.

To emulate the diverse border defects encountered in scanned road engineering drawings—such as broken lines, blurring, and scanning artifacts—we introduce a Multiple-Random Erasing strategy. This method synthesizes highly variable degraded samples, reducing reliance on manual annotations while enhancing the model’s robustness. It works as follows:

1. Random Point Selection: For each table image, first locate all the non-background (characters, box lines) pixels that are manually annotated with or without the help of edge detection. A certain number of pixels are then randomly selected from them to serve as the initial erasure seed points.
2. Random Region Size: For each erasure seed point, we randomly assign a side length within certain range (e.g., from 12 to 24 pixels) formulating the rectangular region to be erased, which is centered on the seed point.
3. Random Pixel Erase: for each region to be erased, we generate a random number for each non-background pixel within the region. By setting a threshold of 0.2, pixels with random numbers greater than the threshold are erased with Gaussian smoothing.

### 3.2. TableBorderNet Architecture

While the SelfSynth Training strategy introduces resilience to diverse degradation patterns by augmenting training data with realistic structural perturbations, the design of the segmentation architecture remains equally critical for robust table border extraction. In particular, the complex spatial layout of engineering tables—often defined by long, thin, and regularly spaced borderlines—demands a network capable of capturing both fine-grained local details and the broader structural organization. Conventional architectures with limited receptive fields or insufficient topological modeling capacity may fail to preserve the continuity and alignment of these linear structures, especially in the presence of visual noise or structural discontinuities. To meet these challenges, a topology-preserving segmentation architecture is developed to explicitly account for the spatial regularity and hierarchical dependencies inherent in tabular layouts.

#### 3.2.1. Encoder: Multi-Scale Feature Extraction

We construct TableBorderNet based on the Fully Convolutional Networks (FCN) [25]. The encoder undertakes the key tasks of feature extraction and hierarchical processing. The input image with a size of 3 × 960 × 960 first undergoes a 7 × 7 convolution operation (stride = 2), followed by batch normalization, the ReLU activation function, and max pooling, adjusting the image size to 64 × 240 × 240. Subsequently, ResNet’s layer1−layer4 are introduced for feature extraction, ultimately converting the feature size to 512 × 30 × 30 and achieving hierarchical extraction of features from shallow to deep layers. Additionally, after the output of layer4, the SliceConv Enhancement module is employed to further enhance feature representation capability and spatial information utilization.

#### 3.2.2. Decoder: Spatial Resolution Reconstruction and Topology Completion

The decoder aims to restore the image size while integrating multi-scale information to achieve accurate predictions. It consists of four DecoderBlocks, each following a processing pipeline of “compression-upsampling-feature integration”. The input features first undergo channel reduction via 1 × 1 convolution, followed by upsampling through a 3 × 3 transposed convolution (stride = 2) to restore spatial resolution. The channel number is then adjusted using 1 × 1 convolution, with each convolution followed by batch normalization and the ReLU activation function. The output of each DecoderBlock is fused with the features from the corresponding upper layer in the encoder as the input for the next DecoderBlock. Finally, the output of the fourth DecoderBlock is passed through a deconvolution layer to restore the feature size to 32 × 960 × 960. A final 3 × 3 convolution and a 1 × 1 convolution layer compress the channels to the number of categories, and the Sigmoid activation function generates pixel-level prediction probability maps, realizing an accurate mapping from abstract semantic features to the original spatial resolution.

#### 3.2.3. SliceConv Enhancement

**Figure 5:**
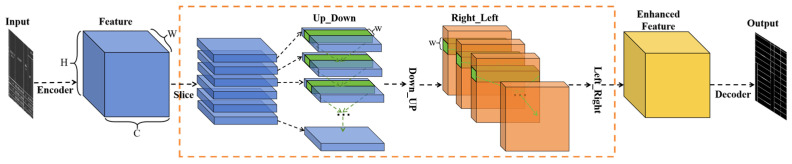
SliceConv Enhancement Module.

To address the strong spatial structural priors of table borders in engineering drawings, we introduce a Topology Feature Enhancement Module, as shown in the orange dashed box in Figure 5. Table borders in road engineering drawings exhibit highly regular spatial structures, appearing as elongated elements spanning across large portions of the image. They are arranged in horizontal, vertical, or diagonal directions with consistent spacing, exhibiting strong spatial regularities and global dependencies. Traditional convolutional networks, however, rely on fixed-size local receptive fields, failing to capture the long-range spatial regularity of table borders. While increasing the convolution kernel size or network depth can expand the receptive field, this often leads to higher computational complexity without necessarily effectively capturing the global spatial information.

To address this limitation, the SliceConv operates directly on the spatial regularity of the feature maps, progressively propagating information along the rows and columns in a top-down, bottom-up, left-to-right, and right-to-left manner. This convolution scheme enables information to regularly flow between pixels within the feature maps, effectively capturing the table border’s topological structural priors and strengthening the network’s ability to express tabular features even with lots of discontinuity at the structural lines, without significantly increasing computational complexity.

Specifically, let the input feature map have dimensions of (C, H, W), where C, H, and W represent the number of channels, rows, and columns, respectively. We first divide the feature map along the channel dimension into C slices, each with a size of (1, H, W). For the first slice, we apply a 1 × 1 convolution to extract initial feature information. To aggregate the topologically regular features within each channel, we then add the convolved first slice to the second slice, updating the second slice’s features. This process continues iteratively, propagating information from the top to the bottom, until the last slice is updated. By propagating information in this manner, the SliceConv enhances the network’s ability to perceive the global spatial structure of table borders, overcoming the limitations of traditional CNNs in global spatial modeling. The specific calculation Equation (1) is as follows:

(1) $$F_{i,j,k}^{\prime }=\left\{\begin{array}{ll} F_{i,j,k}, & j=1\\ F_{i,j,k}+f\left(\sum_{m}\;\sum_{n}\;F_{m,j-1,k+n-1}^{\prime }K_{m,i,n}\right), & j=2,\;3,...H \end{array}\right.,$$

where $F_{i,j,k}$ denotes the feature value at the position with channel number i, row number j, and column number k; $F_{i,j,k}^{\prime }$ denotes the updated feature value; $K_{m,i,n}$ denotes the convolution weight between the elements in channel m and the elements in channel i with an n column offset in the current slice; f is the activation function, and the weights of the convolution kernel are shared across all slices.

### 3.3. Topology-Aware Loss Function

While the SelfSynth Training module enhances robustness to diverse degradation patterns and the TableBorderNet architecture emphasizes structural preservation, the intrinsic imperfections commonly found in scanned engineering drawings—such as broken lines, blurred strokes, and interference from overlapping annotations—pose persistent challenges for topologically consistent table border extraction. These defects often disrupt the continuity of border structures in ways that conventional supervision strategies fail to address effectively.

In particular, the widely used loss, which emphasizes pixel-wise classification accuracy, lacks the capacity to capture higher-level structural dependencies, making it insufficient for guiding the network toward complete and coherent border predictions. To overcome this limitation, the loss function is extended by incorporating a topology-aware component, as shown in Equation (2). By jointly optimizing conventional pixel-level loss ($L_{BCE}$) and the proposed topology-level loss ($L_{TOPOLOGY}$), the network is encouraged not only to improve pixel-level precision but also to restore structural integrity in the presence of local ambiguities, thereby achieving more reliable and topologically faithful segmentation results.

(2) $$L=L_{BCE}+\alpha \cdot L_{TOPOLOGY}.$$

The binary cross-entropy loss ($L_{BCE}$) serves as the foundational loss component, measuring the pixel-level difference between the network’s predictions and the ground truth labels, as shown in Equation (3), where N is the number of samples, $y_{i}^{*}$ is the predicted value for the i-th sample, and $y_{i}$ is the corresponding ground truth label for the i-th sample.

(3) $$L_{BCE}\left(y,y^{*}\right)=-\frac{1}{N}\sum_{i=1}^{N}\left[y_{i}\log\left(y_{i}^{*}\right)+\left(1-y_{i}\right)\log\left(1-y_{i}^{*}\right)\right].$$

While $L_{BCE}$ effectively drives the network to produce accurate pixel-level classifications, it is insufficient for ensuring topological integrity, particularly in challenging scenarios. Thus, we design the topology loss to guide the network in learning the overall topological structure of table borders. This is achieved by ingeniously framing the topology completion problem as a minimization of the difference between the network’s prediction and a target “ideal” difference map.

**Figure 6:**
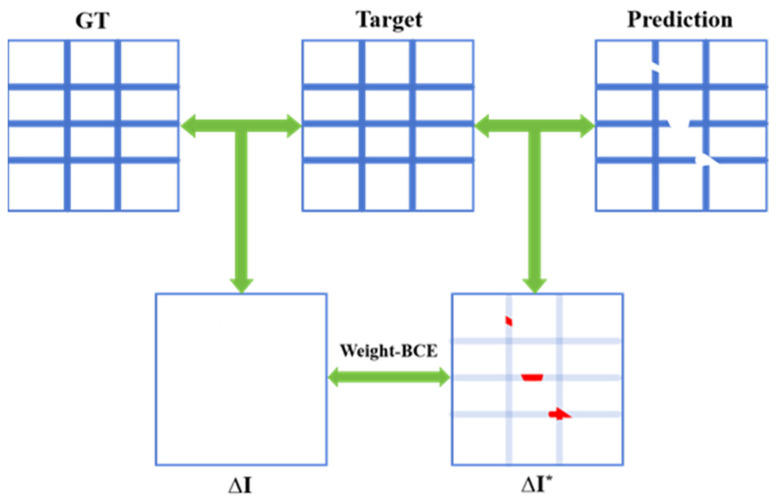
Topology Loss: Arrows denote the comparison between the two, and red contents mark the areas with discrepancies.

As illustrated in Figure 6, we first compute a difference map $\Delta I_{i}^{*}$ by subtracting the network’s i-th prediction from the i-th ground truth (GT). The non-zero pixels in this difference map directly correspond to topological defect areas where the network’s prediction deviates from the ground truth topology. The key insight is that the ideal output of the network would result in a zero-valued difference map ΔI, representing perfect topological agreement with the ground truth. We then assign higher loss weights to these defective regions to steer the network towards producing topologically complete table border segmentation results, as shown in Equation (4), where N is the number of samples, $\Delta I_{i}^{*}$ is the value at the i-th pixel in the difference map. This defect-aware weighting effectively prioritizes the correction of topological errors over pixel-level accuracy in non-defective regions, leading to more robust and visually pleasing table structure extraction.

(4) $$L_{TOPOLOGY}\left(\Delta I^{*}\right)=-\frac{1}{N}\sum_{i=1}^{N}\;\left[\log\left(1-\Delta I_{i}^{*}\right)\right].$$

## 4. Experiments

### 4.1. Dataset and Self-Supervised Training Strategy

The engineering drawing dataset used in this work consists of RGB images with a resolution of 4950 × 3500 pixels, compiled according to standardized drafting formats. To enable automated dataset generation and annotation with minimal manual effort, we propose a generative self-supervised learning strategy to train TableBorderNet.

We first selected 17 engineering drawing images with intact table borders and used traditional edge detection methods (e.g., Canny) to generate initial ground-truth border masks, which were then manually verified for accuracy. Leveraging the global consistency of table structures, where local border patterns closely resemble the overall grid layout, we performed 300 random crops of 960 × 960 regions per image. This yielded a total of 5100 local table patches, with corresponding ground-truth border masks cropped from the same regions to serve as labels.

The 5100 degraded table patches, paired with their clean ground-truth border masks, were split into training (60%, 3060 patches), validation (20%, 1020 patches), and test (20%, 1020 patches) sets. During training, the degraded patches serve as inputs, with the complete border masks as targets, enabling TableBorderNet to learn simultaneous border extraction and defect completion, overcoming the limitations of conventional approaches in handling incomplete tables.

This self-supervised data generation strategy effectively expands the training dataset and introduces realistic table border defects, allowing TableBorderNet to learn robust feature representations and generalize well to diverse real-world engineering drawings.

TableBorderNet is trained using the AdamW optimizer [26] with a weight decay of 0.01 and a batch size of 16. The initial learning rate is set to 1 × 10^−3^ and reduced by a factor of 0.1 after every 30 epochs. Training is conducted for a total of 100 epochs, with early stopping implemented based on the validation loss to prevent overfitting. Specifically, if the validation loss does not decrease for 10 consecutive epochs, training is terminated. The AdamW optimizer was selected due to its ability to effectively handle weight decay, leading to improved generalization performance. The chosen batch size represents a trade-off between memory constraints and training efficiency. Furthermore, we employ gradient clipping with a maximum L2 norm of 1.0 to stabilize training and prevent gradient explosion.

The loss function used for training is a combination of binary cross-entropy loss ($L_{BCE}$) and a topology-aware loss ($L_{TOPOLOGY}$), as described in Section 3.3. The weighting factor factor α for $L_{TOPOLOGY}$ is set to 1, balancing the contribution of both loss terms.

It is important to note that we did not employ traditional data augmentation techniques such as random rotations, horizontal/vertical flips, or scaling. This decision was based on the observation that the fundamental structure of tables in engineering drawings exhibits a high degree of invariance to these transformations. The core challenge lies not in recognizing rotated or scaled versions of table elements, but rather in robustly extracting borders and completing missing segments in the presence of noise and topological defects. Therefore, we prioritized a self-supervised data generation strategy (described in Section 4.1), which focuses on simulating realistic table border degradation and topological disruptions. This approach allows the network to learn directly from the types of imperfections it is likely to encounter in real-world engineering drawings, leading to more effective and targeted training.

### 4.2. Evaluation Metrics

To comprehensively assess the performance of TableBorderNet, we employ a combination of geometric detail metrics and topological structure metrics.

#### 4.2.1. Geometric Detail Metrics

Since table border extraction is formulated as a semantic segmentation task, we evaluate the network’s performance using standard metrics: Precision, Recall, F1-score, and Intersection-over-Union (IoU). These metrics are calculated as follows:

(5) $$\mathrm{Precision}=\frac{\mathrm{TP}}{\mathrm{TP}+\mathrm{FP}},$$

(6) $$\mathrm{Recall}=\frac{\mathrm{TP}}{\mathrm{TP}+\mathrm{FN}},$$

(7) $$F1=\frac{2\times \mathrm{Precision}\times \mathrm{Recall}}{\mathrm{Precision}+\mathrm{Recall}},$$

(8) $$\mathrm{IoU}=\frac{\mathrm{TP}}{\mathrm{TP}+\mathrm{FN}+\mathrm{FP}},$$

where TP (true positive) represents the number of pixels correctly identified as table borders, FP (false positive) denotes the number of pixels incorrectly classified as borders, and FN (false negative) indicates the number of border pixels that were missed by the model.

#### 4.2.2. Topological Structure Metrics

**Figure 7:**
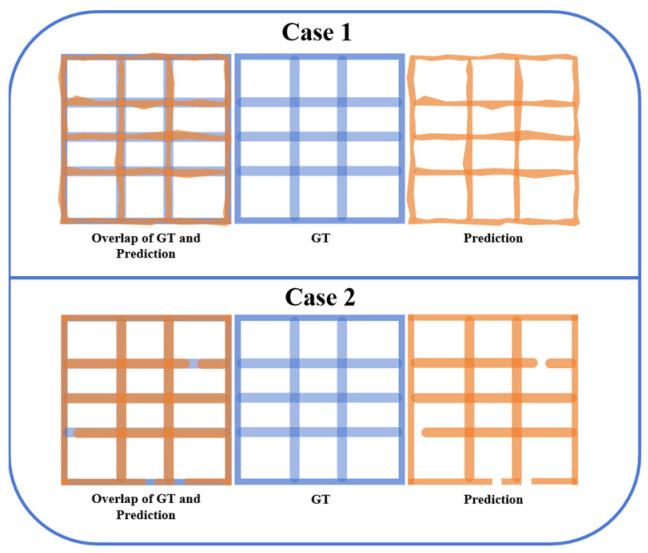
Comparison Between Predicted Border and the Ground Truth.

While geometric detail metrics offer valuable insights into the pixel-level accuracy of border extraction, they fail to fully capture the critical topological properties of table borders, such as connectivity and grid continuity. Consider two illustrative scenarios (Figure 7).

Case 1: Low IoU, Correct Topology. The predicted borders may exhibit a relatively low IoU with the ground truth due to minor geometric inaccuracies, yet they accurately preserve the underlying connected domain structure of the table grid. In such cases, the extracted table remains topologically valid.

Case 2: High IoU, Incorrect Topology. Conversely, the predicted borders may achieve a high IoU with the ground truth, but minor connectivity errors result in merged domains, disrupting the overall topological structure. These seemingly minor pixel-level errors can drastically alter the table’s usability.

Geometric metrics alone can therefore provide a misleading evaluation of the true quality of the extracted table borders, particularly concerning the preservation of essential topological characteristics. For example, a high IoU may mask a topologically incorrect table, while a low IoU might undervalue a topologically sound extraction.

**Figure 8:**
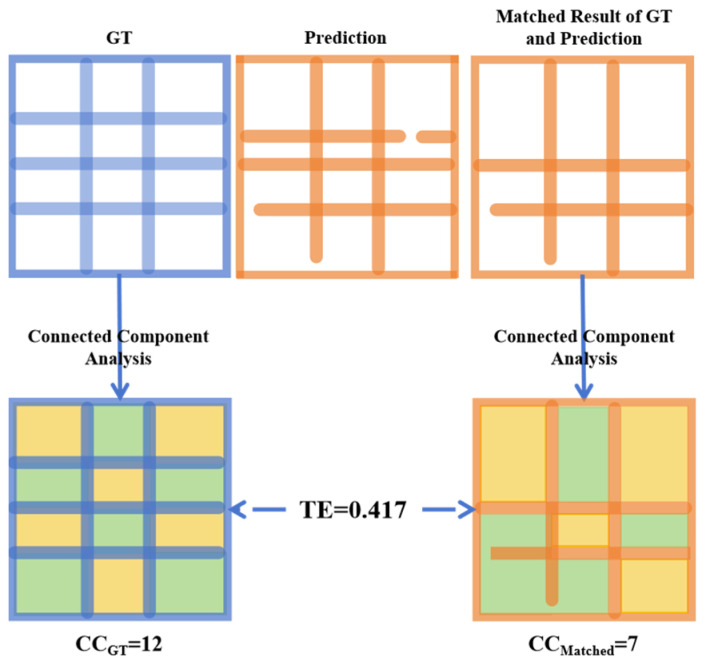
Topology Structure. Different colors of adjacent blocks denote the absence of connectivity between regions, while the same color denotes the presence of connectivity.

To address this limitation, we introduce the Topology Error (TE) metric, which quantifies the connectivity errors based on the numbers of connected domains in the matched predicted and ground-truth borders. As shown in Figure 8, TE is calculated as follows:

(9) $$TE=\frac{\left|{CC}_{{GT}_{i}}-{CC}_{{Matched}_{i}}\right|}{{CC}_{{GT}_{i}}},$$

where ${CC}_{Matched}$ is the number of connected domains in the matched predicted and ground-truth borders, and ${CC}_{GT}$ is the number of connected domains in the ground-truth borders.

By incorporating both geometric detail metrics and the topology-aware TE metric, we can provide a comprehensive evaluation of TableBorderNet’s performance, assessing both the pixel-level accuracy and the preservation of critical topological properties of the extracted table borders.

### 4.3. Results

#### 4.3.1. Table Border Extraction and Completion

TableBorderNet was trained under the following configurations: GPU—NVIDIA RTX 3090, CPU—Intel(R) Xeon(R) CPU—E5-2680 v4, Ubuntu 20.04, CUDA 11.7, and PyTorch 1.13.1. Tests were conducted on the dataset, and we observed that the model effectively segmented borders across various table conditions.

(1) Overall Table Border Extraction

**Figure 9:**
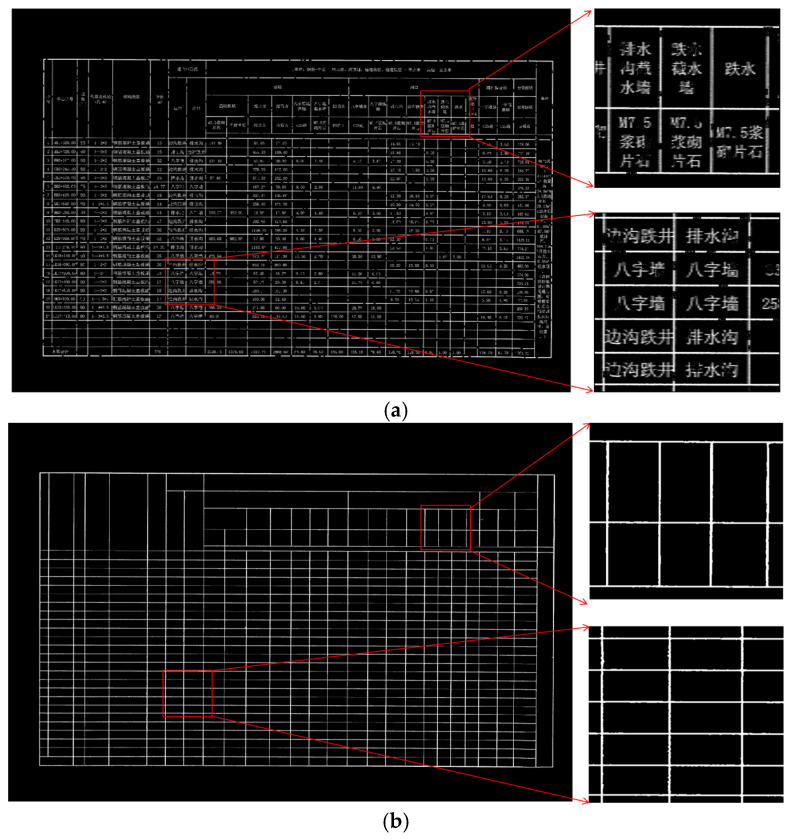
General Table Border Extraction. The Chinese characters in the table are all professional terms related to road engineering. (**a**) Displays the Table Image. (**b**) Depicts the BorderEextraction Result.

As shown in Figure 9, for the input table drawing image, the red-framed area at the lower left exhibits an issue where characters are adherent to box lines, while the red-framed area at the upper right presents unclear box lines. The table as a whole contains dense discontinuities; however, the network can effectively predict complete box lines despite these challenges.

(2) Detailed Table Border Extraction and Completion

**Figure 10:**
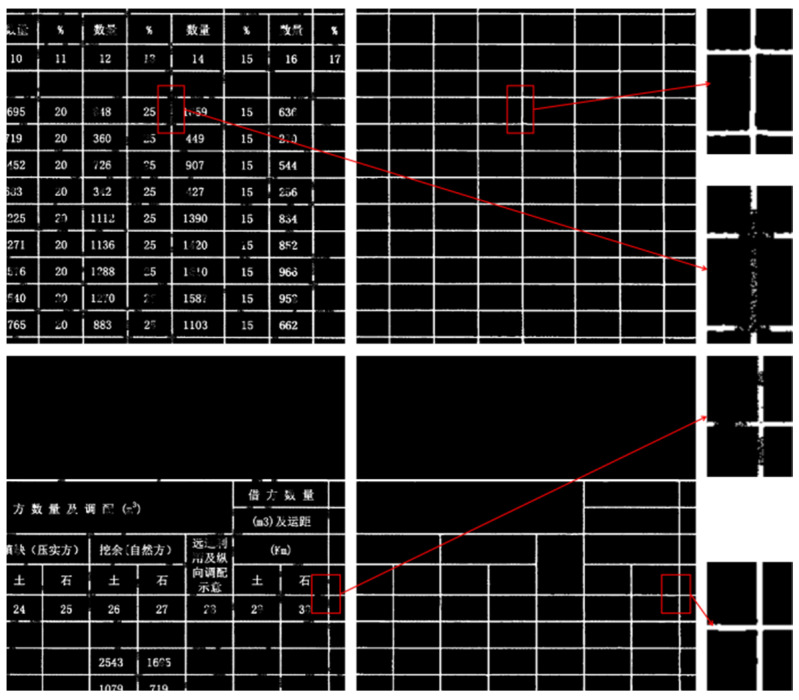
Detailed Table Extraction. The Chinese characters in the table are all professional terms related to road engineering.

As shown in Figure 10, the table borders have clear delineations. The tables in the first row are relatively simple, with a structured and uniform layout of rows and columns, without any prominent merged cells. The text distribution is also evenly spaced, resulting in an overall clean appearance. In contrast, the tables in the second row contain multiple merged cells, especially in the header section, and some cells have dense text content. For the extraction results on the right, TableBorderNet successfully ignores the text pixels and extracts the corresponding table borders. It also effectively handles dense missing borders and restores the topological connectivity of the extracted lines, even in the intersections of the rows and columns.

(3) Fuzzy Table Border Extraction and Completion

**Figure 11:**
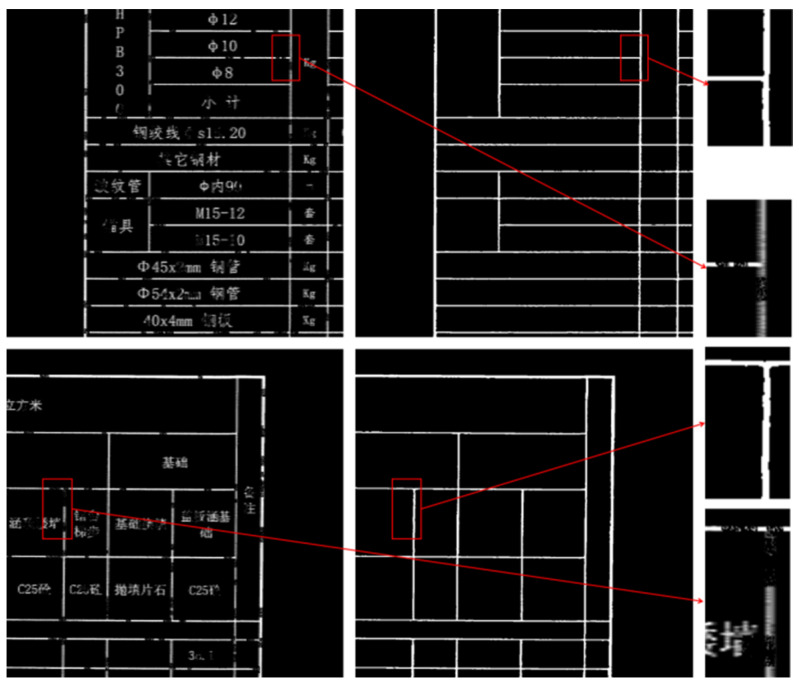
Fuzzy Table Border Extraction. The Chinese characters in the table are all professional terms related to road engineering.

Figure 11 presents tables where the borders exhibit low local contrast with the surrounding regions, making the edges less distinct. The table layouts are more diverse, with greater variations in cell dimensions. Due to cell merging, some vertical borders do not extend across the entire table, appearing as multiple segments at the same vertical position with varying lengths and distribution patterns. TableBorderNet’s extraction results for these fuzzy table borders are binarized images, effectively enhancing the contrast between the borders and the background. Even for the severely degraded regions, close to complete erasure, shown in the zoomed-in area of the second row, the network successfully restores the borders without discarding the short, discontinuous border segments at the image boundaries, maintaining the overall table structure.

(4) Character-Adjoined Table Border Extraction and Completion

**Figure 12:**
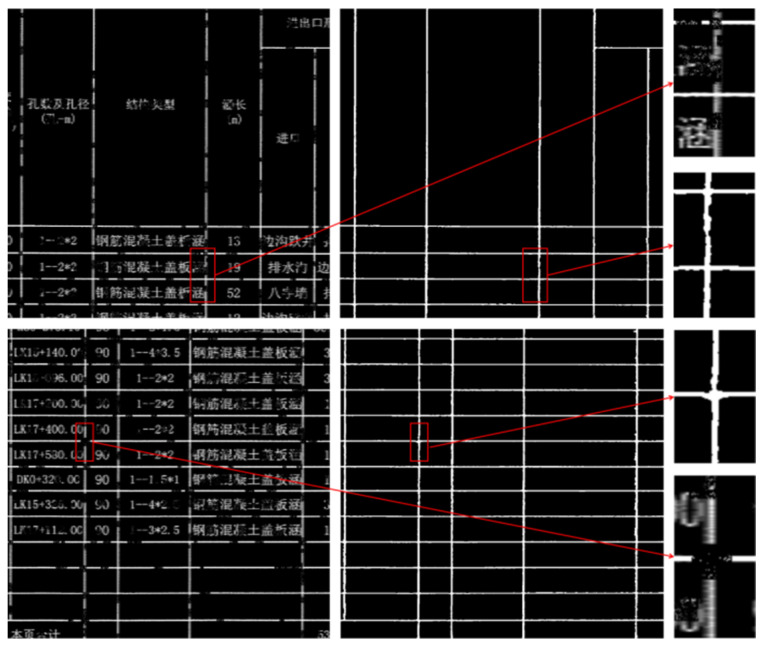
Extraction of Table Borders with Character Adjoining. The Chinese characters in the table are all professional terms related to road engineering.

Figure 12 shows tables with a relatively organized layout, but some cells contain densely packed text. In the first row, the characters are directly connected to the fuzzy borders, and due to the random nature of the simulated defects, both the connected characters and borders exhibit degradation, increasing the difficulty of border completion. The tables in the second row have richer textual content within the cells, where the characters are in close proximity to the unclear border edges. Additionally, there is a vertical border at the left edge of the image, which typically lacks sufficient contextual information. Nevertheless, TableBorderNet accurately extracts the table borders, effectively distinguishing between cell content and border lines, and avoiding text interference. Furthermore, for the mixed defect regions in the first row, the network successfully restores the connectivity of the discontinuous borders, and the border at the left edge of the second row image is also completely preserved.

As shown in Table 1, for the semantic segmentation-based table border extraction, Precision represents the proportion of true positives among the samples predicted as positive. TableBorderNet’s Precision of 96.1% indicates that it effectively avoids misclassifying background or non-border regions as borders. Recall measures the network’s ability to successfully identify all positive samples, reflecting the model’s sensitivity to borders. TableBorderNet’s Recall approaching 98% suggests that it comprehensively identifies all table borders and can effectively complete missing border regions. The F1-score, which is the harmonic mean of Precision and Recall, reaches 97% for TableBorderNet, demonstrating the network’s stability in ensuring low misclassification while also minimizing missed detections. The IoU, which calculates the overlap between the predicted and ground-truth border regions, reaches 94.2% for TableBorderNet, indicating its ability to extract table borders with high precision.

Furthermore, we compared TableBorderNet against LDCNet [27], a model that is dedicated to addressing the discontinuity of drawings. It cascades a ResNet [28]-based semantic segmentation network and a classic line defect detection and repair network. The results show that while maintaining comparable Precision, TableBorderNet outperforms LDCNet in Recall, F1, and IoU, indicating its superior ability to capture and accurately extract table borders, especially in tasks that require comprehensive border detection, such as processing complex tables or noisy environments. The borders predicted by TableBorderNet also more closely align with the actual table border boundaries. In addition, we conducted experiments using the recent semantic segmentation architecture ConDSeg [29], along with the widely used classical architectures U-Net [30] and DeepLab v3+ [31]. The experimental results indicate that our method achieves superior performance in handling this type of task.

Overall, TableBorderNet demonstrates a robust understanding of table structures, capable of handling complex table layouts, particularly those with merged cells, dense text characters, and irregular arrangements. It also exhibits strong adaptability to the diverse row–column structures and content distributions within tables. Moreover, TableBorderNet can successfully extract table borders even when the input images contain noise or blur, showcasing its high robustness. Even when text overlaps with the lines in the tables, the model can still accurately identify and distinguish the borders.

#### 4.3.2. Effectiveness of Topology Loss

To validate the impact of the topology loss function on the extraction of topologically complete table borders, we conducted comparative experiments on TableBorderNet using the traditional binary cross-entropy loss (BCE Loss) and the topology-aware loss (BCE Loss + Topo Loss). The analysis of the connectivity error rate (TE) and the changes in the number of connected domains further demonstrated the effectiveness of the topology loss function.

As shown in Table 2, the model trained with only the BCE loss exhibited a connectivity error rate of 1.548%, while the introduction of the topology loss significantly reduced the error rate to 1.070%. This result indicates that the topology loss function substantially enhances the network’s learning capacity regarding the topological structure of table borders, effectively reducing the topology error rate.

**Figure 13:**
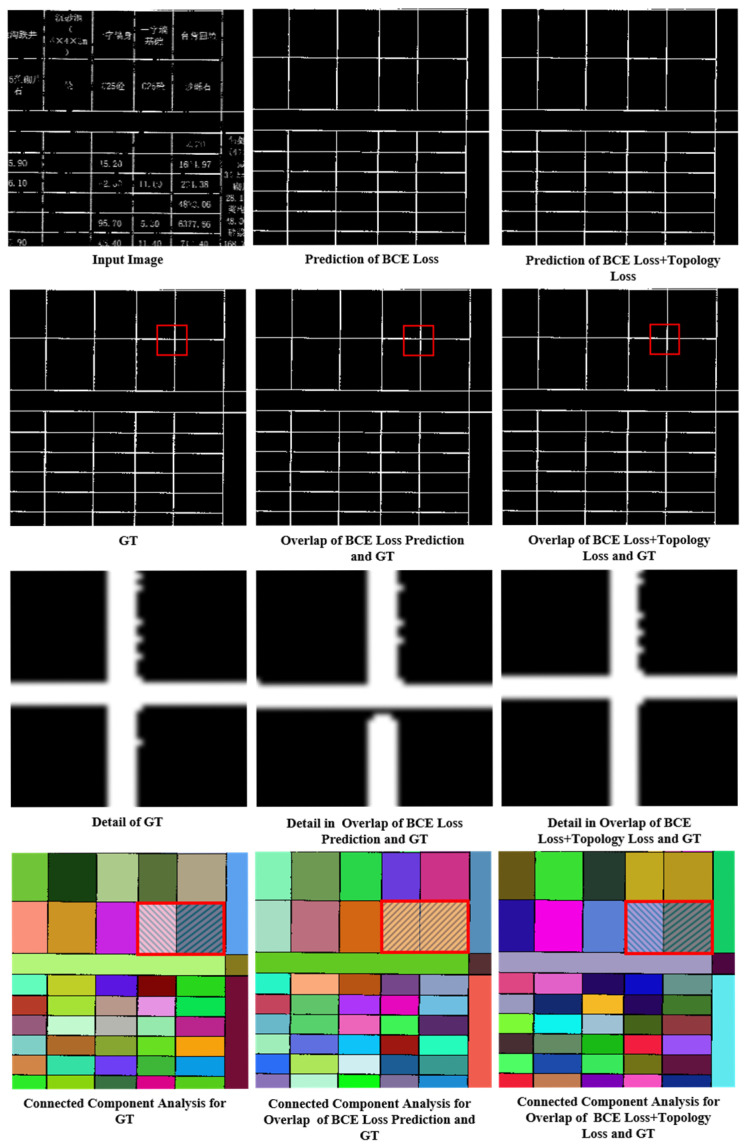
Visulization Comparison of Loss Function: In the first row, the Chinese characters in the table are all professional terms related to road engineering. The images in the third row are derived from the regions marked by the red frames in the second-row images. In the fourth row, different colors of adjacent blocks denote the absence of connectivity between regions, while the same color denotes the presence of connectivity.

The visualization results in Figure 13 further illustrate this. The first row shows the degraded input image, the prediction using only the BCE loss, and the prediction with the added topology loss. The second row displays the ground truth, the BCE prediction matched with the ground truth, and the topology loss prediction matched with the ground truth. The third row provides magnified details, revealing that the BCE prediction fails to fully complete the missing pixels at the intersections of the borders. The fourth row presents the connected domain analysis, where the BCE prediction results in the merging of adjacent domains due to the lack of critical border pixels. In contrast, the topology loss function effectively guides the network to learn the global topological structure of the table borders, making it more sensitive to structural defects and enabling accurate restoration of discontinuous borders, even in challenging regions.

Compared to using only the BCE loss, where the network can extract relatively complete table borders and automatically complete most defective regions, the topology loss helps address the missing critical pixels that, although few in number, are crucial for the topological connectivity of the borders. The topology loss function effectively directs the network to learn the overall topological structure of the table borders, allowing it to be more sensitive to topological defects and better restore and complete the discontinuous borders, maintaining high-precision topological structure recovery even in regions with border breaks or blurriness.

## 5. Conclusions and Future Work

This paper presents TableBorderNet, a network specifically designed for the extraction of table borders from scanned road engineering drawings. By expanding the receptive field and incorporating global topological structure learning, TableBorderNet significantly enhances the accuracy of table border extraction and the connectivity of the extracted structures. The design of the topology loss function further strengthens the model’s ability to complete missing border regions, leading to a reduction in the topology error rate.

Overall, TableBorderNet excels in both pixel-wise segmentation accuracy and global structural awareness. Its performance metrics are impressive, achieving an IoU of 94.2% and a TE of 1.07% on a dataset of 5100 degraded patches derived from 17 original clean tables. The topology loss function reduces TE from 1.548% (using only BCE loss) to 1.07%, indicating a substantial improvement in the network’s capacity to learn the topological structure of table borders, effectively lowering the error rates associated with connectivity.

In practical applications, TableBorderNet significantly reduces the reliance on manual annotations, requiring only 17 initial tables compared to the typical 500–1000 needed for conventional supervised methods—this represents a 30–60× reduction in labeling effort. Additionally, the model cuts manual processing time from 15 min to just 10 s per table, yielding a 90-fold efficiency gain. It also achieves impressive accuracy in budget table data attribution, surpassing LDCNet’s performance. These gains highlight TableBorderNet’s transformative potential for engineering digitization, with applications extending to financial balance sheets, medical records, and other structured data systems.

Despite its strengths, TableBorderNet has limitations. The self-supervised approach relies on synthetic degradation, which may not fully replicate real-world variability, such as handwritten notes or non-uniform scans. Additionally, irregular layouts with non-grid structures or extensive cell merging challenge its assumptions of regular row–column patterns.

Future research can enhance TableBorderNet in several key areas:

(1) Efficiency Optimization: Refining the topology feature enhancement module using lightweight architectures (e.g., MobileNet) to reduce computational overhead while maintaining accuracy.
(2) Robustness Improvement: Employing generative adversarial networks (GANs) to simulate diverse real-world degradation patterns, improving adaptability to severe damage and organic variability.
(3) Generalization Expansion: Integrating multimodal learning (text, borders, semantics) via transfer learning to handle non-standard tables (e.g., nested or handwritten layouts), broadening applicability across domains like finance and healthcare.

By balancing technical innovation with practical utility, TableBorderNet establishes a new benchmark for automated table extraction, offering a scalable and accurate solution with the potential to transform structured data management across various fields. The findings indicate significant research value and application potential for future advancements in engineering, finance, medical, and other domains.

## Figures and Tables

**Table 1:** Performance of TableBorderNet.

Model	Precision	Recall	F1	IoU
LDCNet [27]	0.962	0.971	0.966	0.935
ConDSeg [28]	0.974	0.846	0.905	0.827
Unet [29]	0.961	0.882	0.920	0.852
Deeplab v3+ [30]	0.999	0.871	0.931	0.870
TableBorderNet (ours)	0.961	0.979	0.970	0.942

**Table 2:** Comparison of Loss Function.

Model	Loss	TE
TableBorderNet	BCE Loss	1.548%
TableBorderNet	BCE Loss+Topology Loss	1.070%

## Data Availability

Data are contained within the article.

## Abbreviations

The following abbreviations are used in this manuscript:

OCR	Optical Character Recognition
TE	Topological Error
IoU	Intersection over Union
R-CNN	Region-based Convolutional Neural Networks
FCN	Fully Convolutional Networks

## References

[1] Chen H., Zhu Y., Luo X. (2022). TableGraph: An Image Segmentation–Based Table Knowledge Interpretation Model for Civil and Construction Inspection Documentation. J. Constr. Eng. Manag..

[2] Ali B., Khusro S. A Divide-and-Merge Approach for Deep Segmentation of Document Tables. Proceedings of the 10th International Conference on Informatics and Systems.

[3] Luo D., Peng J., Fu Y. Biotable: A tool to extract semantic structure of table in biology literature. Proceedings of the 5th International Conference on Bioinformatics Research and Applications.

[4] Hasan F., Kashevnik A. (2022). Intelligent Frame and Table Segmentation in Blueprint Documents: Method and Implementation. Proceedings of the Computational Methods in Systems and Software.

[5] Zou Y., Ma J. A deep semantic segmentation model for image-based table structure recognition. Proceedings of the 2020 15th IEEE International Conference on Signal Processing (ICSP).

[6] Wang H., Xue Y., Zhang J., Jin L. (2022). Scene table structure recognition with segmentation collaboration and alignment. Pattern Recognit. Lett..

[7] Ballard D. (1981). Generalizing the Hough transform to detect arbitrary shapes. Pattern Recognit..

[8] Ma C., Lin W., Sun L., Huo Q. (2022). Robust Table Detection and Structure Recognition from Heterogeneous Document Images. Pattern Recognit..

[9] Hashmi K.A., Stricker D., Liwicki M., Afzal M.Z. (2021). Guided Table Structure Recognition Through Anchor Optimization. IEEE Access.

[10] Raja S., Mondal A., Jawahar C.V. (2020). Table structure recognition using top-down and bottom-up cues. Proceedings of the Computer Vision–ECCV 2020: 16th European Conference.

[11] Siddiqui S.A., Fateh I.A., Rizvi S.T.R., Dengel A., Ahmed S. Deeptabstr: Deep learning based table structure recognition. Proceedings of the IEEE 2019 International Conference on Document Analysis and Recognition (ICDAR).

[12] Xue W., Li Q., Tao D. Res2tim: Reconstruct syntactic structures from table images. Proceedings of the IEEE 2019 International Conference on Document Analysis and Recognition (ICDAR).

[13] Siddiqui S.A., Khan P.I., Dengel A., Ahmed S. Rethinking semantic segmentation for table structure recognition in documents. Proceedings of the IEEE 2019 International Conference on Document Analysis and Recognition (ICDAR).

[14] Long R., Wang W., Xue N., Gao F., Yang Z., Wang Y., Xia G.-S. Parsing table structures in the wild. Proceedings of the IEEE/CVF International Conference on Computer Vision.

[15] Koci E., Thiele M., Romero O., Lehner W. A genetic-based search for adaptive table recognition in spreadsheets. Proceedings of the IEEE 2019 International Conference on Document Analysis and Recognition (ICDAR).

[16] Tensmeyer C., Morariu V.I., Price B.L., Cohen S.D., Martinez T.R. Deep splitting and merging for table structure decomposition. Proceedings of the IEEE 2019 International Conference on Document Analysis and Recognition (ICDAR).

[17] Déjean H., Meunier J.-L. Table rows segmentation. Proceedings of the IEEE 2019 International Conference on Document Analysis and Recognition (ICDAR).

[18] Khan S.A., Khalid S.M.D., Shahzad M.A., Shafait F. Table structure extraction with bi-directional gated recurrent unit networks. Proceedings of the IEEE 2019 International Conference on Document Analysis and Recognition (ICDAR).

[19] Qiao L., Li Z., Cheng Z., Zhang P., Pu S., Niu Y., Ren W., Tan W., Wu F. (2021). Lgpma: Complicated table structure recognition with local and global pyramid mask alignment. Proceedings of the International Conference on Document Analysis and Recognition.

[20] He K., Gkioxari G., Dollár P., Girshick R. Mask r-cnn. Proceedings of the IEEE International Conference on Computer Vision.

[21] Paliwal S.S., D V., Rahul R., Sharma M., Vig L. Tablenet: Deep learning model for end-to-end table detection and tabular data extraction from scanned document images. Proceedings of the IEEE 2019 International Conference on Document Analysis and Recognition (ICDAR).

[22] Shelhamer E., Long J., Darrell T. (2016). Fully convolutional networks for semantic segmentation. IEEE Trans. Pattern Anal. Mach. Intell..

[23] Nguyen D.-D. (2021). TableSegNet: A fully convolutional network for table detection and segmentation in document images. Int. J. Doc. Anal. Recognit. (IJDAR).

[24] Pang L., Zhang Y., Ma C., Zhao Y., Zhou Y., Zong C. (2024). TableRocket: An Efficient and Effective Framework for Table Reconstruction. Proceedings of the Chinese Conference on Pattern Recognition and Computer Vision (PRCV).

[25] Wang J., Song L., Li Z., Sun H., Sun J., Zheng N. End-to-end object detection with fully convolutional network. Proceedings of the IEEE/CVF Conference on Computer Vision and Pattern Recognition.

[26] Kingma D.P., Ba J. (2014). Adam: A method for stochastic optimization. arXiv.

[27] Sasaki K., Iizuka S., Simo-Serra E., Ishikawa H. Joint gap detection and inpainting of line drawings. Proceedings of the IEEE Conference on Computer Vision and Pattern Recognition.

[28] He K., Zhang X., Ren S., Sun J. Deep residual learning for image recognition. Proceedings of the IEEE Conference on Computer Vision and Pattern Recognition.

[29] Lei M., Wu H., Lv X., Wang X. (2025). ConDSeg: A General Medical Image Segmentation Framework via Contrast-Driven Feature Enhancement. Proc. AAAI Conf. Artif. Intell..

[30] Ronneberger O., Fischer P., Brox T. (2015). U-net: Convolutional networks for biomedical image segmentation. Medical Image Computing and Computer-Assisted Intervention–MICCAI 2015: 18th International Conference, Munich, Germany, 5–9 October 2015.

[31] Chen L.C., Zhu Y., Papandreou G., Schroff F., Adam H. Encoder-decoder with atrous separable convolution for semantic image segmentation. Proceedings of the European Conference on Computer Vision (ECCV).

